# Weight increase in people with cystic fibrosis on CFTR modulator therapy is mainly due to increase in fat mass

**DOI:** 10.3389/fphar.2023.1157459

**Published:** 2023-07-13

**Authors:** Marialena Mouzaki, Annie Dupuis, Julie Avolio, Katherine Griffin, Felix Ratjen, Elizabeth Tullis, Tanja Gonska

**Affiliations:** ^1^ Department of Pediatrics, Cincinnati Children’s Hospital Medical Center, Cinncinati, OH, United States; ^2^ Dalla Lana School of Public Health, University of Toronto, Toronto, ON, Canada; ^3^ Department of Pediatrics, University of Toronto, Hospital for Sick Children, Toronto, ON, Canada; ^4^ Department of Medicine, University of Toronto and St. Michael’s Hospital, Toronto, ON, Canada

**Keywords:** cystic fibrosis, ivacaftor treatment, nutritional status, body composition, resting energy expenditure trail registration: NCT03390985 BMI-body mass index, CFTR modulator treatment

## Abstract

**Background:** Ivacaftor, the first CFTR modulator drug, leads to significant long-term improvement in lung function and weight gain. The mechanism as well as the long-term impact of ivacaftor on weight, resting energy expenditure (REE) and body composition remains to be explored.

**Methods:** This prospective observational study included 18 people with CF (pwCF) (age: median (range) 20 (6–58) years) carrying at least one CFTR gating mutation commencing ivacaftor. Assessments of body composition, REE and laboratory investigations were performed at baseline and 6, 12 and 24 months after treatment initiation.

**Results:** Treatment with ivacaftor was associated with a significantly positive change in BMI z-score at 24 months. Fat mass (mean (95% CL) of 6.5 kg (4.0; 9.0) from baseline, *p* = 0.0001), but not fat-free mass changed under ivacaftor treatment. There was a significant positive correlation between weight and fat mass change. Overall, there was no significant change in measured REE from baseline (mean (95% CL) of 108 kcal/d (−12; 228), *p* = 0.07) in our cohort. Pancreatic function and other nutritional markers did not change with treatment, with the exception of an increase in serum vitamin A levels (*p* = 0.006).

**Conclusion:** The weight gain observed in ivacaftor treated pwCF is predominantly secondary to increases in fat mass warranting early counseling of people starting on CFTR-modulating treatment with respect to healthy diet and physical exercise.

## Highlights

• REE in G551D CF on ivacaftor did not change, except in 3 patients with high baseline REE
• Weight increase on ivacaftor treatment was associated with increase in fat mass
• No change in pancreas function.

## Introduction

The treatment of patients with cystic fibrosis (CF) carrying mutation G551D was revolutionized with the introduction of ivacaftor into clinical care (Ramsey et al., 2011). This CFTR modulator drug increases the opening probability of CFTR channels and has been shown to improve lung function and reduce pulmonary exacerbations in patients carrying gating mutations. In addition, the use of ivacaftor has been associated with clinically significant weight gain (Ramsey et al., 2011; Stallings et al., 2016). The mechanism underlying the weight gain seen in this context, however, has not yet been fully elucidated.

PwCF typically have a lower weight, body mass index (BMI), as well as a reduced fat and lean mass compared to their peers (Newby et al., 1990; Bianchi et al., 2006; Sheikh et al., 2014). The altered body composition seen in CF may be secondary to a variety of factors, such as poor intake due to anorexia; inefficient digestion/absorption secondary to pancreatic insufficiency (PI), liver disease and/or intestinal inflammation; as well as increased energy expenditure, which occurs particularly in the context of more advanced lung disease or concurrent infections (Fried et al., 1991; Pencharz and Durie, 2000). In addition, catabolism associated with systemic inflammation can further impact patients’ muscle mass (Ionescu et al., 2002). Interventions, such as nutritional rehabilitation have been shown to lead to improvements in weight (Levy et al., 1985); however, less is known about the impact of dietary modifications on the body composition of these patients. Similarly, little is known about the long term impact of CFTR modulators, such as ivacaftor on body composition (Tierney et al., 2015; Stallings et al., 2018).

The primary objective of our study was to investigate whether the weight gain seen in adults and children with CF receiving treatment with ivacaftor is associated with changes in energy expenditure. Further, we wanted to explore the change in body composition occurring in this context.

## Subjects and methods

This was a prospective observational study performed at the Hospital for Sick Children and at St. Michael’s Hospital in Toronto between 2013 and 2016. The Research Ethics Board of both academic institutions approved the study (SickKids Research Ethics Board (REB)# 1000036224 and SMH REB# 13–089), which was in compliance with the ethical principles outlined in the declaration of Helsinki. All participants and/or caregivers signed an informed consent prior to study enrolment. Minors signed assent forms.

### Patient cohort

PwCF carrying at least one gating CFTR mutation who were commencing treatment with ivacaftor 150 mg twice daily between 2011 and 2016 were included in this observational study. Exclusion criteria were the inability to undergo assessment of body composition (via air displacement plethysmography) or energy expenditure (via indirect calorimetry).

Patients were categorized according to their pancreatic function as: 1. Insufficient (PI) if they had a fecal elastase <100 μg/g stool and/or serum trypsinogen <10 ng/ml; 2. Borderline if they had a fecal elastase ranging between 50 and 200 μg/g stool and a serum trypsinogen >10 ng/ml; and, 3. Pancreatic sufficient (PS) if they had a fecal elastase >200 μg/g stool and/or serum trypsinogen >10 ng/ml.

### Monitoring

Patients were initiated on treatment when clinically stable and data were collected from their routine clinical visits prior to starting ivacaftor (pre-drug), as well as 6 months (post-6; 5.3–7.1 months), 12 months (post-12; 11.7–14.8 months) and 24 months (post-24; 23.5–28.3 months) after starting treatment with ivacaftor. Data collection included anthropometric measurements, measurements of energy expenditure and body composition, and laboratory investigations. Levels of fat-soluble vitamins were only determined annually. Lung function was assessed by spirometry (FEV1 percent predicted [FEV1pp] according to European Respiratory Society/American Thoracic Society standards using Global Lung Initiative equations (Quanjer et al., 2012)), as well as the Lung Clearance Index (LCI) using the multiple breath washout was measured (Subbarao et al., 2015).

### Anthropometric measurements

Weight and height were assessed using a digital scale and a stadiometer mounted on the wall, respectively. Body mass index was calculated as kg/cm^2^. BMI values were converted to z-scores using the World Health Organization references (https://www.who.int/growthref/en/).

### Resting energy expenditure (REE)

Energy expenditure was measured by indirect calorimetry using VMax™ Encore 29 machine (Carefusion Medical Products, Yorba Linda, California) operated by a trained technician. Indirect calorimetry was considered successful and acceptable when the patients reached steady state for a minimum of 10 min and the respiratory quotient was within the acceptable physiologic range (0.67 and 1.35). Predicted REE was calculated using the Food and Agriculture Organization/World Health Organization/United Nations University (FAO/WHO/UNU) equation (Energy and protein requirements, 1985). A measured REE>110% of that predicted by the WHO equation was used to define hypermetabolism.

### Body composition

Body composition assessments were performed using air displacement plethysmography (BOD POD 2007A, Life Measurements Inc.). Patients were asked to wear either tight fitting underwear or a bathing suit and their hair was covered with a tightly fitting cap. The subjects were asked to sit in the chamber and breathe calmly without moving during the measurements. Two 40-s measurements were taken per person; measurements were considered successful when the deviation in body volume was less than 3 mL/L subject size. The average of the two measurements was used in the analyses.

### Laboratory investigations

Laboratory investigations included nutritional markers (e.g., albumin, fat soluble vitamins), markers of pancreatic function (serum trypsinogen, fecal elastase), as well as markers of inflammation, such as C-reactive protein and absolute neutrophil count.

### Statistical analyses

Data across the clinic visits were modeled in two ways: 1) observed value across all visits and 2) the change from baseline at each follow-up visit. The mixed-effects model for repeated measurements (MMRM; PROC MIXED; SAS statistical software package, SAS Institute, Cary, NC) was used to control for the within subject correlation of the repeated measures across visits. Changes from baseline controlled for baseline values as a covariate in the model. We tested the effect of pancreatic status by adding it to the model of change from baseline, and we tested the effect of age and change in weight in the models of REE across visits and change from baseline. Results are provided as estimates plus 95% confidence limits (CI). The primary outcome was change in REE and the secondary outcome were change in body composition. We also analyzed the change in other variables, which in total can be grouped into 7 broad categories (including REE, body composition, anthropometric measures, lung function, inflammation markers, pancreatic function status and nutritional markers). Therefore, we set the level of significance at 0.05/7 = 0.007. Lastly, we calculated the Pearson correlation between the change in REE and other variables for the change between baseline visit and the post-6 months visit, as most of the changes happened during this time frame.

## Results

Eighteen pwCF were included in this study; 7 subjects were younger than 17 years of age at the time of the baseline visit (Table 1). The majority of the pwCF (n = 12) were pancreatic insufficient (PI), 3 pwCF were pancreatic sufficient (PS) and 3 had fecal elastase and serum trypsinogen measurements in the borderline range. For the purpose of the analyses, pwCF with a borderline pancreas function were grouped with the PS patients.

**TABLE 1:** Baseline characteristics of study cohort.

Variable		Result
≤17 years (n = 11)	mean (range)	10.6 (6.1–14.9)
>17 years (n = 7)	mean (range)	33.8 (17.8–58.3)
Gender - female	n (%)	11 (61%)
Genotype	(n)	G551D/F508del (12), G178R/F508del (1)
		G551D/2622 + 1G>A (1), G551D/E585X (1)
		G551D/3272-26A>G (2), G551D/unknown (1)
Pancreatic insufficiency	n (%)	12 (67%)
CF-related diabetes	n (%)	1 (6%)

### Anthropometrics

**FIGURE 1:**
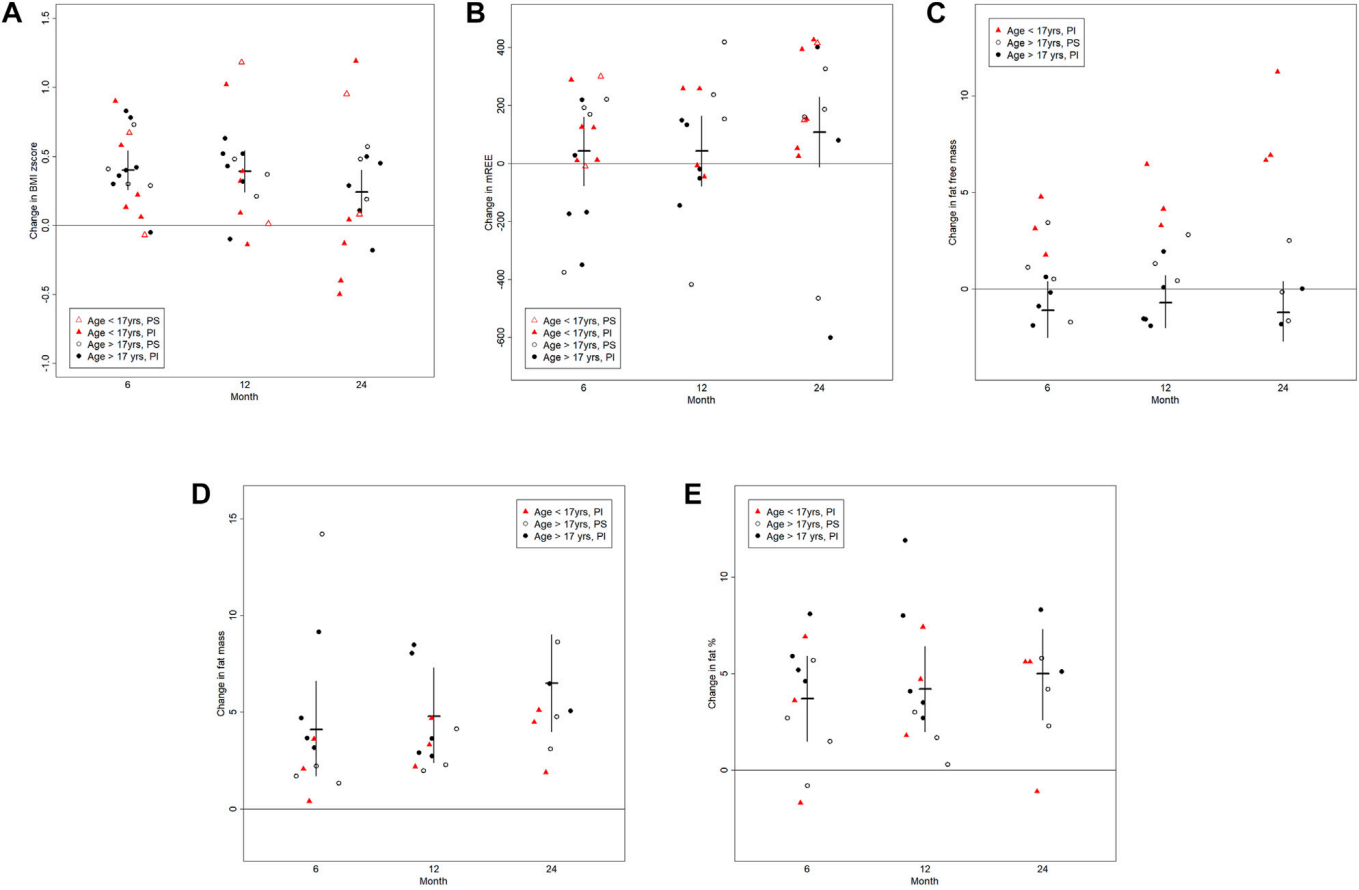
The graphs shows changes in the **(A)** body mass index (BMI, kg/cm^2^), **(B)** resting energy expenditure (REE (kcal/d)), **(C)** fat free mass (kg), **(D)** fat mass (kg) and **(E)** fat percent (%) between baseline and follow-up visits at post-6, post-12 and post-24 months. Results are visualized as scattered dot plots reflecting individual results. The horizontal line represents the mean and the vertical lines represent 95% confidence limits.

Over the 24-month treatment period all pwCF demonstrated an increase in BMI z-score (*p* = 0.003). This change in anthropometrics occurred in the first 6 months of treatment, with no significant changes thereafter (Figure 1A and Table 2). One pwCF with PS was removed from the post-12 and post-24 analyses because this patient underwent gastric bypass surgery. The changes in anthropometric measures were independent of the pancreas function status (pancreatic status effect on change in weight, *p* > 0.9).

**TABLE 2:** Summary data of the clinical visits before and after patients started on ivacaftor.

	Repeated measures of values at each visit model controls for age at baseline				Repeated measures of change from visit 1 (baseline); model controls for baseline value and age at baseline		
	Baseline	Post-6	Post-12	Post-24	Post-6—Baseline	Post-12—Baseline	Post-24—Baseline
	*Estimate (n)*				*Estimate (95%CL) p-value*		
	*95%CL*						
**Primary Outcome**							
mREE/kcal/day	1466 (18)	1500 (16)	1513 (13)	1576 (14)	42 (−77; 160)	43 (−79; 164)	108 (−12; 228)
	1347; 1585	1378; 1623	1384; 1643	1449; 1703	0.5	0.5	0.076
mREE/kcal/day/kg	30.3 (18)	28.5 (16)	27.6 (12)	27.1 (13)	−1.8 (−3.7; 0.2)	−2.9 (−5.0;-0.8)	−3.1 (−5.2;-1.1)
	27.3; 33.2	25.5; 31.5	24.4; 30.7	23.9; 30.2	0.075	0.009	0.005
mREE, % predicted	111.4 (18)	110.3 (16)	102.9 (12)	104.7 (13)	−1.5 (−9.5; 6.6)	−8.3 (−17.7; 1.1)	−6.0 (−15.0; 3.1)
	102.2; 120.6	100.7; 120.0	91.9; 113.8	94.1; 115.3	0.7	0.080	0.18
**Secondary Outcomes**							
**Anthropometric Measures**							
BMI z-score	0.50 (18)	0.90 (18)	0.88 (16)	0.73 (15)	0.40 (0.26; 0.54)	0.39 (0.24; 0.54)	0.24 (0.09; 0.40)
	0.05; 0.94	0.46; 1.34	0.43; 1.33	0.29; 1.18	<.0001	<.0001	0.003
**Body Composition (fat)**							
Fat mass (kg)	12.3 (13)	16.5 (11)	16.8 (11)	18.3 (8)	4.1 (1.7; 6.6)	4.8 (2.4; 7.3)	6.5 (4.0; 9.0)
	5.8; 18.8	10.0; 23.1	10.3; 23.3	11.7; 24.9	0.003	0.0007	0.0001
Fat mass (kg)^a^	6.7 (10)	11.6 (8)	11.5 (8)	13.7 (5)	5.7 (−0.6; 12.1)	6.3 (−0.1; 12.6)	8.6 (2.2; 14.9)
	−2.9; 16.4	1.9; 21.4	1.8; 21.3	3.8; 23.5	0.072	0.052	0.013
Fat %	21.1 (13)	25.0 (11)	25.7 (11)	26.4 (8)	3.7 (1.5; 5.9)	4.2 (2.0; 6.4)	5.0 (2.6; 7.3)
	14.9; 27.4	18.7; 31.3	19.4; 32.0	20.1; 32.8	0.003	0.0008	0.0004
Fat %^a^	15.1 (10)	19.4 (8)	19.6 (8)	21.5 (5)	4.7 (0.3; 9.0)	4.9 (0.6; 9.1)	6.9 (2.5; 11.2)
	6.4; 23.9	10.5; 28.2	10.8; 28.5	12.6; 30.4	0.037	0.030	0.0056
**Body Composition (lean)**							
Fat free mass (kg)	42.5 (13)	43.2 (11)	43.9 (11)	45.0 (8)	0.6 (−1.1; 2.3)	1.2 (−0.4; 2.9)	2.3 (0.5; 4.1)
	34.9; 50.1	35.5; 50.8	36.2; 51.5	37.3; 52.7	0.5	0.12	0.01
Fat free mass (kg)^a^	50.6 (10)	50.6 (8)	50.8 (8)	50.2 (5)	0.0 (−1.3; 1.3)	0.2 (−1.1; 1.6)	−0.4 (−2.0; 1.1)
	40.1; 61.2	40.0; 61.2	40.3; 61.4	39.6; 60.8	>0.9	0.7	0.5

^a^ Age at baseline >17 years, mREE-measured resting energy expenditure.

### Resting energy expenditure

Measurements of the REE were available for all 18 pwCF at baseline, for 16 pwCF 6 months post treatment initiation as well as for 13 and 14 pwCF at 12 and 24 months post treatment initiation, respectively. At baseline 7 pwCF were hypermetabolic. With respect to the entire cohort, there was no significant change in the ratio of measured to predicted REE or clinically significant change in the absolute REE over the treatment course of months (Figure 1B and Table 2). However, the 3 pwCF with the highest measured to predicted REE ratio of 136%, 186% and 146% at baseline showed a decrease to 103%, 108% and 100% at 24 months post drug (Supplementary Figure 1A).

### Body composition (fat free mass)

Data on body composition were available for 13 pwCF at pre-drug assessment and for 11 pwCF 6 months post drug, as well as for 11 and 8 pwCF at 12 and 24 months post drug assessment. There was no significant change in fat free mass over the observation time, except for the 3 children ≤17 years of age for which we had body composition data (difference post-6 months to baseline (age)): 4.8 kg (12), 3.1 kg (15) and 1.8 kg (16)) (Figure 1C).

### Body composition (fat mass)

In contrast to the fat free mass, fat mass increased by a mean (95%CL) of 6.5 kg (4.0; 9.0), *p* = 0.0001 24 months post drug. The change in fat mass was independent of the pancreatic function status (*p* = 0.8) and occurred in both children and adults (Figure 1D and Table 2).

Given the above, treatment was associated with an overall mean increase of 5 percent body fat (95%CL (2.7; 7.3), *p* = 0.0004) by 24 months) (Figure 1E and Table 2). Individual increase in fat mass correlated with increase in body weight (Supplementary Figure 1B).

### Pancreatic function

There was no significant change in pancreatic function measured by fecal elastase and serum trypsinogen over the course of the study (Table 3). In pwCF with PI, fecal elastase remained <100 μg/g stool and in those with PS, fecal elastase remained >200 μg/g stool. The three pwCF classified as having a borderline pancreatic function remained in the borderline range over the 24-month period.

**TABLE 3:** Summary data of pancreas status, lung function and inflammation markers before and after patients started on ivacaftor.

	Repeated measures of values at each visit				Repeated measures of change from baseline visit model controls for baseline value				
	Baseline	Post-6	Post-12	Post-24	Post-6–Baseline	Post-12–Baseline		Post-24–Baseline	
	*Estimate (n)*				Estimate (95%CL) *p*-value				
	95%CL								
**Pancreas status-Insufficient**									
Trypsinogen (ng/mL)	3.54 (11)	2.79 (10)	2.59 (8)	4.80 (3)	−0.88 (−1.44;-0.32)		−0.91 (−1.51;-0.31)		1.43 (0.37; 2.50)
	2.86; 4.23	2.08; 3.49	1.79; 3.39	3.53; 6.07	0.0043		0.0054		0.011
Fecal Elastase (μg.g fat)^*^	15.6 (8)	16.3 (9)	14.4 (7)	19.9 (5)					
	10.9; 20.3	11.8; 20.8	9.4; 19.3	13.9; 26.0					
**Pancreas status-Sufficient**									
Trypsinogen (ng/mL)	34.5 (6)	16.3 (6)	31.4 (5)	30.5 (3)	−16.6 (−42.9; 9.8)		0.1 (−26.9; 27.2)		−6.7 (−35.6; 22.2)
	16.2; 52.9	−2.1; 34.6	12.0; 50.7	7.8; 53.2	0.2		>0.9		0.6
Fecal Elastase (μg.g fat)^*^	234 (3)	159 (3)	259 (3)	209 (3)					
	82; 386	13; 306	111; 407	58; 360					
**Nutritional markers**									
Vitamin A (μmol/L)	1.32 (17)		1.52 (16)	1.65 (13)			0.21 (0.01; 0.40)		0.33 (0.12; 0.54)
	1.13; 1.52		1.32; 1.72	1.42; 1.88			0.04		0.006
Vitamin E (μmol/L)	19.6 (17)		21.6 (17)	22.8 (13)			2.2 (−1.2; 5.7)		3.7 (−0.1; 7.4)
	16.0; 23.3		18.0; 25.2	18.8; 26.8			0.19		0.057
Vitamin D (μmol/L)	77 (14)		88 (17)	84 (12)			14.3 (1.7; 26.9)		2.7 (−11.2; 16.6)
	62; 93		74; 103	68; 100			0.030		0.7
Albumin (g/L)	42.6 (17)		43.0 (17)	42.8 (13)			0.2 (−1.0; 1.4)		0.3 (−1.2; 1.7)
	41.5; 43.6		41.9; 44.0	41.6; 44.1			0.7		0.7
**Inflammation markers**									
CRP (mg/L)	4.1 (18)		2.0 (14)	1.6 (10)			−1.5 (−3.2; 0.1)		−2.5 (−4.4;-0.7)
	2.5; 5.6		0.3; 3.8	−0.5; 3.7			0.064		0.016
WBC (9 × 10^9^/L)	8.8 (18)	7.7 (15)	7.6 (17)	7.4 (15)	−1.1 (−2.2;-0.1)		−1.2 (−2.2;-0.2)		−1.4 (−2.5;-0.3)
	7.6; 10.1	6.4; 9.0	6.4; 8.9	6.1; 8.7	0.037		0.022		0.011
Neutrophil count (9 × 10^9^/L)	5.9 (10)	4.1 (9)	4.5 (11)	3.9 (10)	−1.9 (−3.1;-0.6)		−1.8 (−3.1;-0.5)		−2.0 (−3.4;-0.7)
	4.6; 7.1	2.8; 5.5	3.3; 5.7	2.6; 5.1	0.005		0.009		0.005
**Lung function**									
FEV1, % predicted	72.1 (18)	85.2 (18)	86.4 (17)	84.0 (16)	13.3 (8.0; 18.6)		14.6 (9.1; 20.0)		12.2 (6.7; 17.7)
	63.5; 80.7	76.6; 93.8	77.7; 95.0	75.3; 92.7	<.0001		<.0001		<.0001
FVC, % predicted	90.6 (18)	101.3 (18)	103.1 (17)	97.9 (14)	10.8 (7.3; 14.3)		12.6 (9.1; 16.2)		7.4 (3.6; 11.1)
	84.9; 96.4	95.6; 107.1	97.3; 109.0	91.9; 103.9	<.0001		<.0001		0.0003
LC2.5	13.3 (11)	11.2 (11)	11.1 (10)	11.1 (5)	−2.4 (−3.2;-1.5)		−2.5 (−3.4;-1.5)		−2.5 (−3.8;-1.3)
	11.1; 15.6	9.0; 13.5	8.9; 13.4	8.7; 13.5	<.0001		<.0001		0.0003
**Other**									
Sweat Chloride (mmol/L)	83 (18)	43 (11)	36 (16)	38 (15)	−41 (−47;-34)		−46 (−53;-40)		−44 (−50;-37)
	77; 88	35; 50	30; 42	32; 45	<.0001		<.0001		<.0001

Change model does not control for baseline. Grey shaded areas indicate that the results do not meet the adjusted significant level of *p* = 0.007 (see METHOD).

### Biochemical nutritional markers

Except for one pwCF with low vitamin A, D and E levels at baseline, which normalized during the follow-up period, no other patient had fat-soluble vitamin deficiencies on regular vitamin supplementation. There was no change in vitamin D and E levels with treatment, but we observed an increase in serum vitamin A levels (*p* = 0.006; Table 3). Most of the pwCF (9/12) in whom vitamin A levels increased had also been on vitamin A supplementation.

### Lung function and inflammation

Treatment with ivacaftor was associated with an improvement in FEV1pp (*p* = 0.0001) and a reduction in the lung clearance index (*p* = 0.0003) in 2 years. Furthermore, we observed a trend in declining serum C-reactive protein levels (*p* = 0.016) and a statistically significantly decrease in the absolute neutrophil count levels (*p* = 0.005) (Table 3).

**FIGURE 2:**
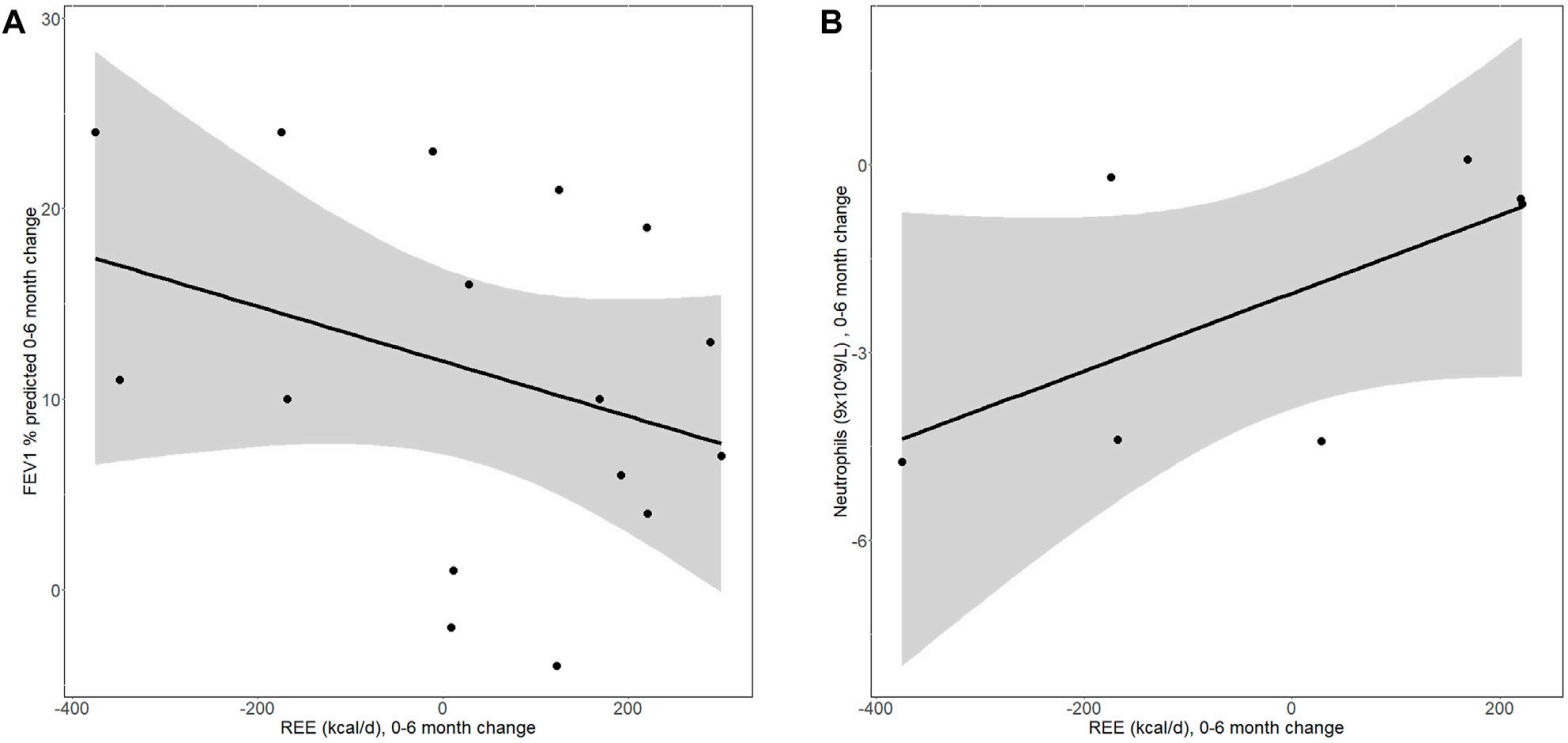
The graphs show correlation between the post-6 months change in **(A)** REE (kcal/d) and FEV1pp and **(B)** REE (kcal/d) and neutrophil count (9 × 10^9^/L). Dots reflect individual results. Line represents Pearson correlation and the grey shaded area the 95%confidence limit. **(A)** *r* ^2^ = −0.33 (−0.69; 0.16), *p* = 0.2, n = 16) and **(B)** *r* ^2^ = 0.64 (−0.04; 0.92), *p* = 0.12, n = 7). Tough the sample was too small to reach significance, the trend/direction of these correlations are interesting.

We next wanted to see whether the change on weight or REE correlated with change in lung function or inflammation. There was no correlation between change in weight and lung function or inflammatory markers. However, while statistical significance was not reached mainly due to small numbers, we observed a negative correlation between REE and lung function (*r* ^2^ = −0.33 (−0.69; 0.16), *p* = 0.2) as well as positive correlation between REE and neutrophil count in sputum (*r* ^2^ = 0.64 (−0.04; 0.92), *p* = 0.12) (Figure 2).

## Discussion

In this prospective, observational study we have shown that the weight gain seen following ivacaftor treatment in children and adults with CF resulted predominantly from increases in fat mass, which occurred largely in the first 6 months of treatment. Fat free mass did not change significantly with treatment, with the exception of those who were in the pediatric age range at the time of ivacaftor initiation. Weight and fat mass gains were not associated with a decline in REE, nor were they associated with changes in pancreatic function or other serum markers of nutritional status.

At 12 months following treatment start with ivacaftor, 46% of pwCF had excess adiposity compared to 38% at baseline. In contrast to the z-BMI, which showed an initial increase in 6 months and then no further increase, the fat mass as well as the %body fat continue to increase over the 24 months observations. Clinicians should be aware of the excess adiposity seen in these patients, particularly during treatment with CFTR modulator. More importantly, BMI is an inadequate indicator of obesity (Javed et al., 2015), as only two pwCF at baseline assessment and one pwCF at 12 months post-drug had a z-BMI >2. Considering the rates of increased body fatness in these subjects, the association between fat free mass and lung function and the importance of the latter in terms of long-term outcomes, physical activity should be recommended to all children and adults with CF. Further, all pwCF starting on CFTR-modifying treatment should receive dietary consultation aiming for a healthy balanced diet.

Previous studies have reported on weight gain induced by CFTR-modulating treatment, though with a shorter duration of follow-up (Ramsey et al., 2011; Borowitz et al., 2016). One study evaluated change in REE and body composition in pwCF on ivacaftor treatment over an observation period of 3 months (Stallings et al., 2018). In contrast to our findings, Stalling et al. (Stallings et al., 2018) observed an increase in fat free mass and fat mass in pwCF on ivacaftor using dual x-ray absorptiometry (DXA) to assess body composition, with an overall increase in %body fat by 1.7% ± 2.3%. Another study saw no difference in fat mass in a 28d-short term ivacaftor-placebo crossover trial in 20 people carrying at least on G551D mutation using bioelectric impedance analysis to evaluate for body composition changes. However, in King’s cohort fat mass increased in the first 6 months on ivacaftor as did weight without any further increase between 6 and 24 months (King et al., 2021). This is different to our observation showing continuous increase in fat mass and fat% of the body composition over the 2 years.

Discrepancy in findings may be due to differences in the methodologies used to assess body composition, the differences in the follow-up time, as well as differences in the baseline body composition of the cohorts. In our study we chose to use air displacement plethysmography (BOD POD^®^) instead of DXA as we aimed for repeated assessments of body composition over a 2-year period. BOD POD^®^ does not expose patients to radiation, is convenient and well accepted by research participants. Despite the fact that BOD POD^®^ is fairly new compared to DXA, it has been used extensively in both pediatric and adult research, and has been shown to generate results comparable to DXA (Talbert et al., 2009; Gracia-Marco et al., 2012; Campisi et al., 2015; Polfuss et al., 2016).

In our study cohort, there was no significant change in REE when expressed as percent predicted REE. This is different to (Stallings et al., 2018), who showed a change in REE over an observation period of 3 months and a negative correlation between the changes in the ratio of measured to predicted REE and weight gain. As per the authors most of the subjects had measured to predicted REE <100%, which is unusual for pwCF. Our cohort was very heterogeneous with regards to their baseline measured to predict REE. Nevertheless, most of the pwCF showed a decline in their measured to predicted REE%pred following ivacaftor treatment, particularly those 3 patients with highest REE%pred at baseline. Since we did not measure lean body mass, but rather fat free mass using the BOD POD^®^, we cannot provide REE ratios in respect to the individual lean body mass.

There was no improvement in pancreatic function throughout the duration of the study. This may have to do with the age of the pwCF at the time of ivacaftor initiation. According to current belief there is an age threshold in CF upon which the pancreas is likely to be irreversibly damaged due to end organ destruction with fibrous-fatty tissue replacement, though case reports have shown recovery of pancreas function in adolescent patients (Gould et al., 2022). Beyond energy expenditure and pancreatic function, other factors could have contributed to the weight gain seen with treatment, such as improved nutrient absorption and/or changes in appetite. Increased absorption in the context of treatment with ivacaftor could possibly be directly related to improved CFTR function at the level of the intestinal epithelium. It has been shown that ivacafor treatment led to an improvement in intestinal pH in pwCF (Gelfond et al., 2017). Reduction in intestinal inflammation as a consequence of ivacaftor-restored CFTR function can also contribute to improved nutrient absorption (Ooi et al., 2018).

In our cohort, treatment with ivacaftor led to a decrease in markers of systemic inflammation, such as CRP and the absolute neutrophil count, which is a systemic marker of inflammation. There is supportive evidence that the reduction in systemic inflammation may be one of the explanations for the increase in body weight in patients on CFTR-modulating treatment.

Ratjen et al. have shown a correlation between a decrease in systemic inflammation markers and weight gain in Pseudomonas-negative CF patients treated with azithromycin as anti-inflammatory therapy (Ratjen et al., 2012). Association between inflammation and nutritional status, is further supported by the study of (Naon et al., 1993) who had shown that ivacaftor reduced pulmonary inflammatory markers in an observational study of 12 pwCF carrying G551D (Hisert et al., 2017). Recently, reduction in systemic inflammation was shown in 20 pwCF treated with highly efficient CFTR modulator (elexacaftor/tezacaftor/ivacaftor) who also had significant improvement in weight, BMI and nutritional parameters (Carnovale et al., 2022).

However, our sample for analysis was small and the results should be interpreted with caution. Future studies to elucidate the association between systemic inflammation and weight gain may need to focus on those pwCF with low weight z-scores and abnormal REE.

The limitation of the study is the relatively small sample size as we were recruiting only pwCF with G551D starting on the highly efficient CFTR modulator ivacaftor. Another limitation is the paucity of data on dietary intake, as well as physical activity levels, which could have affected weight and body composition changes. Lastly, this was an observational study reflecting “real-life” setting of CFTR-modulation in pwCF. This means that drug compliance was not controlled for.

In summary, we have demonstrated that long-term treatment with ivacaftor is associated with weight gain, which is predominantly driven by an increase in fat mass that occurs in the first 6 months of treatment. Changes in REE and pancreatic function are not responsible for the changes seen in our cohort. A significant proportion of pwCF following ivacaftor treatment has excess adiposity and should receive appropriate counseling regarding lifestyle changes.

## Data Availability

The raw data supporting the conclusion of this article will be made available by the authors, without undue reservation.

## Abbreviations

BMI, Body Mass Index; CF, Cystic Fibrosis; CFTR, Cystic Fibrosis Transmembrane Conductance Regulator; CI, Confidence Interval; DXA, Dual X-ray Absorptiometry; FAO, Food and Agriculture Organization; FEVpp, Forced Expiratory Volume in one second Percent Predicted; LCI, Lung Clearance Index; PI, Pancreatic Insufficiency/Insufficient; PS, Pancreatic sufficient; REE, Resting Energy Expenditure; UNO, United Nations University; WHO, World Health Organization.
