# Supplementary figures and images for: Weight increase in people with cystic fibrosis on CFTR modulator therapy is mainly due to increase in fat mass
Source: Front Pharmacol. 2023 Jul 13;14:1157459. doi: 10.3389/fphar.2023.1157459 (PMC10372433; doi:10.3389/fphar.2023.1157459)

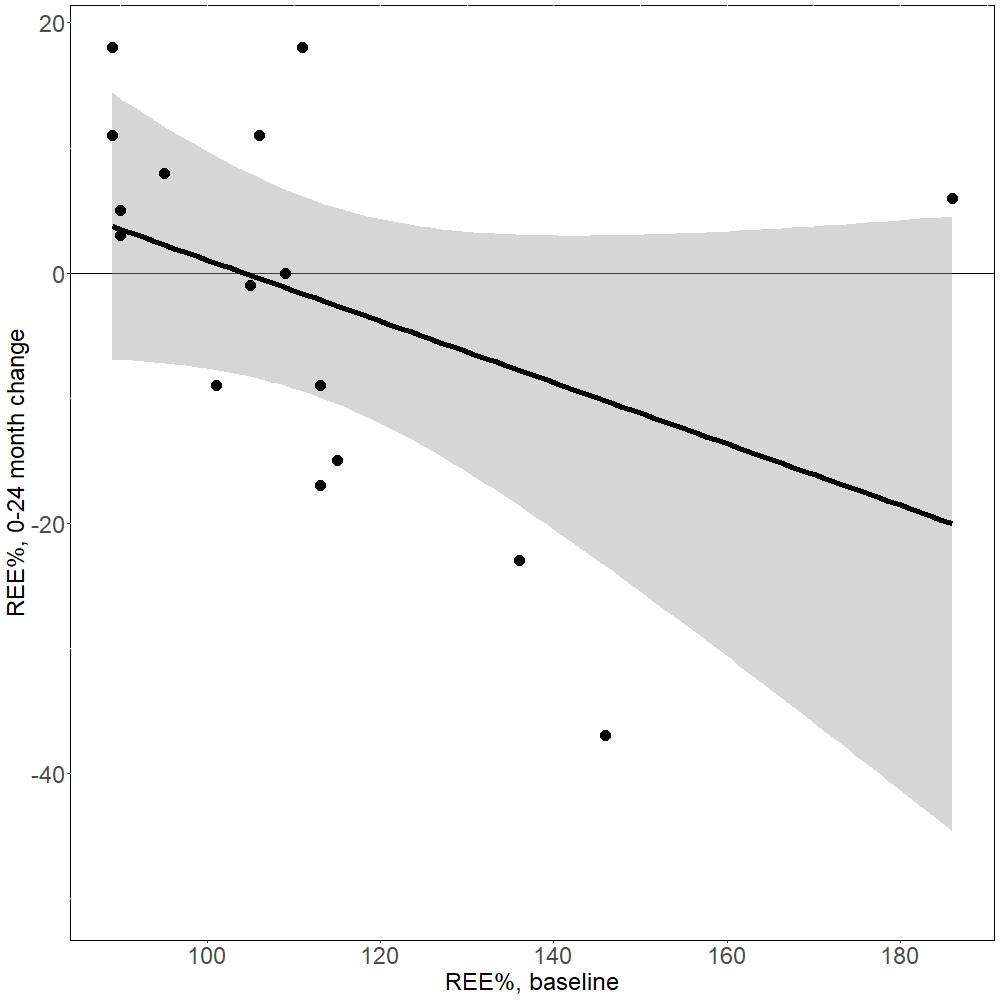

Supplement: Supplementary file 1 [file Image1.JPEG]

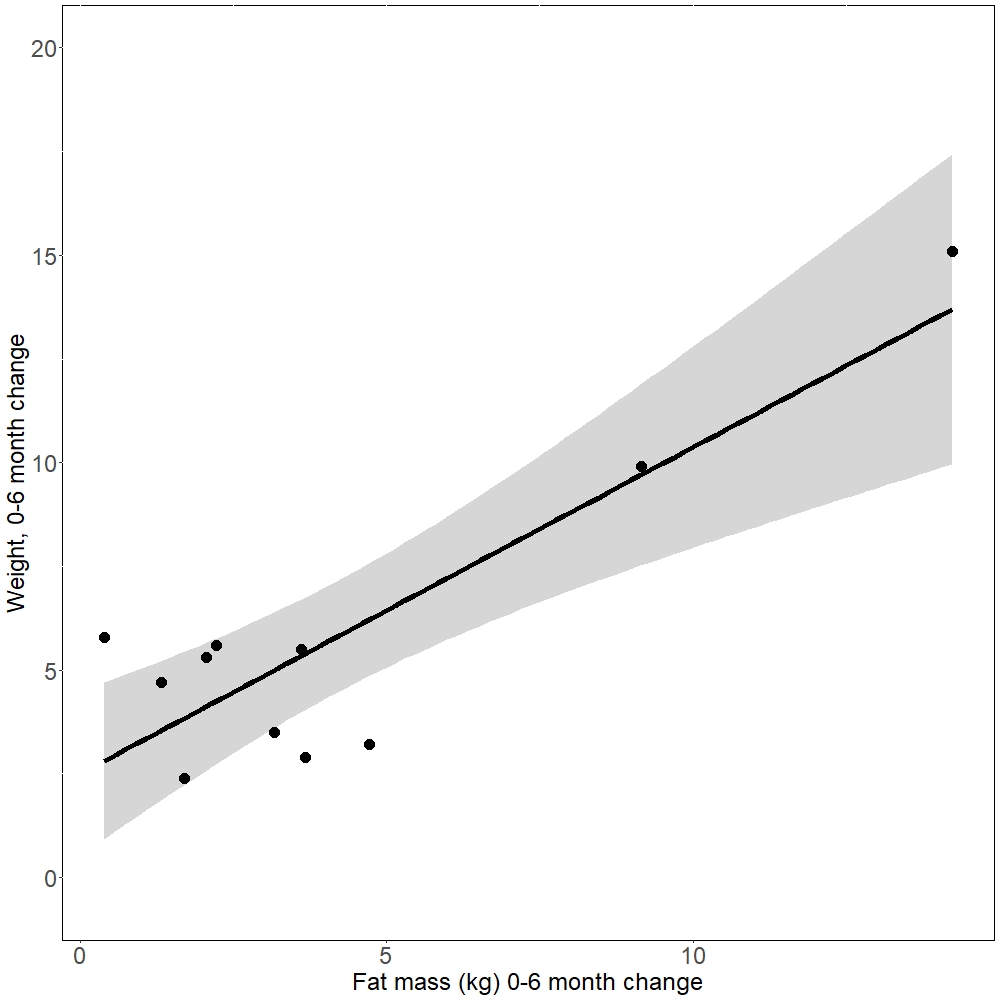

Supplement: Supplementary file 2 [file Image2.JPEG]
