# Supplementary material for: Weight increase in people with cystic fibrosis on CFTR modulator therapy is mainly due to increase in fat mass
Source: Front Pharmacol. 2023 Jul 13;14:1157459. doi: 10.3389/fphar.2023.1157459 (PMC10372433; doi:10.3389/fphar.2023.1157459)
Supplement: Supplementary file 3 [file DataSheet1.PDF]

Legend for Supplement-Figure 1

The graphs show correlation between the post-6 months change in A) REE% to REE% baseline, and B) weight (kg) and change in fat mass (kg). Dots reflect individual results. Line represents Pearson correlation and the grey shaded area the 95%confidence interval. A) r^2= -0.33 (-0.69;0.16), p=0.2, n=16, and B) r^2= 0.86, 95%CL (0.54;0.96), p=0.0006, n=12.
